# Reviewers are fundamental to success of the International Brazilian Journal of Urology

**DOI:** 10.1590/S1677-5538.IBJU.2025.01.02

**Published:** 2025-01-10

**Affiliations:** 1 Universidade do Estado do Rio de Janeiro Unidade de Pesquisa Urogenital Rio de Janeiro RJ Brasil Unidade de Pesquisa Urogenital - Universidade do Estado do Rio de Janeiro - Uerj, Rio de Janeiro, RJ, Brasil; 2 Hospital Federal da Lagoa Serviço de Urologia Rio de Janeiro RJ Brasil Serviço de Urologia, Hospital Federal da Lagoa, Rio de Janeiro, RJ, Brasil

In 2024 the International Brazilian Journal of Urology maintained the impact factor above 3 and this fact was possible because the serious peer review process of our Journal (1). In this year we received more than 650 papers. The Editor-in-Chief would like to thanks all the reviewers and specially to the Doctors: Alexandre Danilovic (Hospital das Clínicas da Faculdade de Medicina da USP -São Paulo, SP, Brasil); Arnold P. Achermann (Universidade Estadual de Campinas - UNICAMP); Daniele Castellani (Ospedali Riuniti di Ancona, Italy); Eduardo Mazzucchi (Universidade de São Paulo - USP, SP, Brasil); Henry H. Woo(Sydney Adventist Hospital Clinical School, ); Evangelos Liatsikos (Hospital of Patras, Greece); José C. Truzzi (Universidade Federal de São Paulo - UNIFESP); José de Bessa (Universidade Estadual de Feira de Santana - UEFS); Ricardo Miyaoka (Universidade Estadual de Campinas - UNICAMP); Wilmar Azal Neto (Universidade Estadual de Campinas - UNICAMP); who reviewed more than 3 articles during the year and strictly within the deadline,

Thanks a lot!!!!!


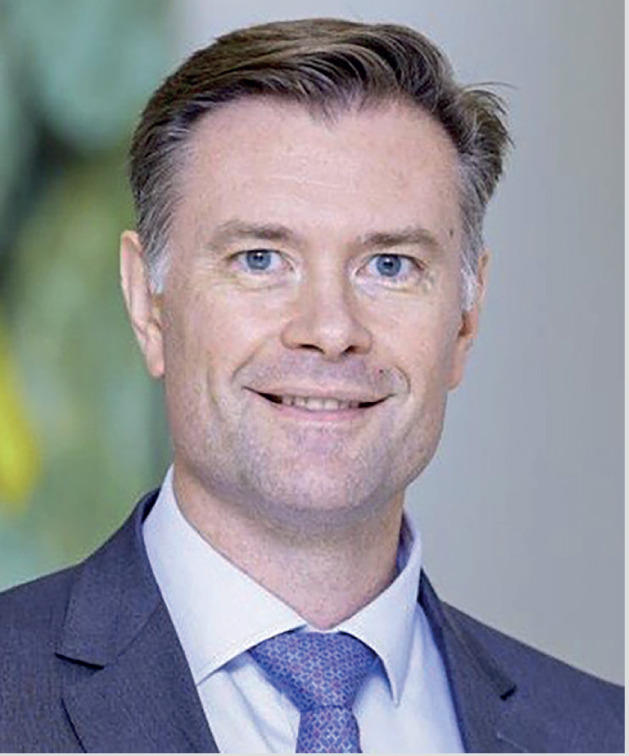


**Alexandre Danilovic**


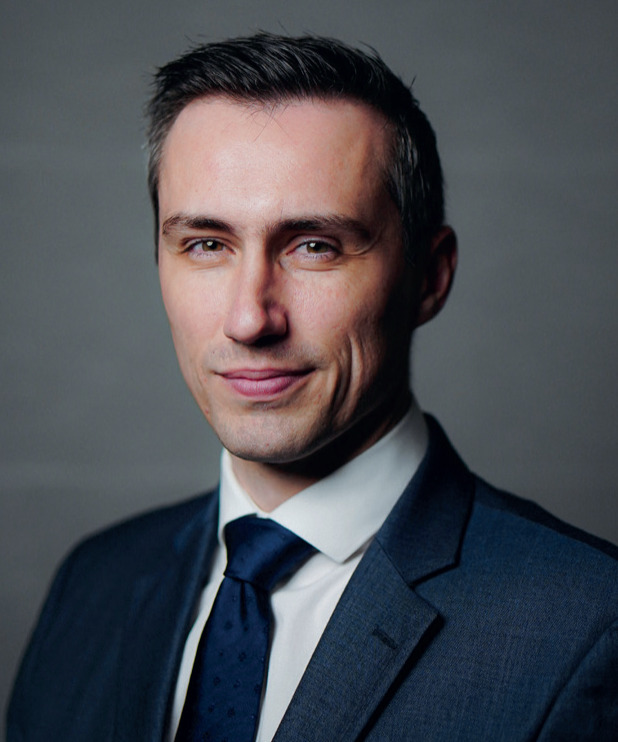


**Arnold Acherman**


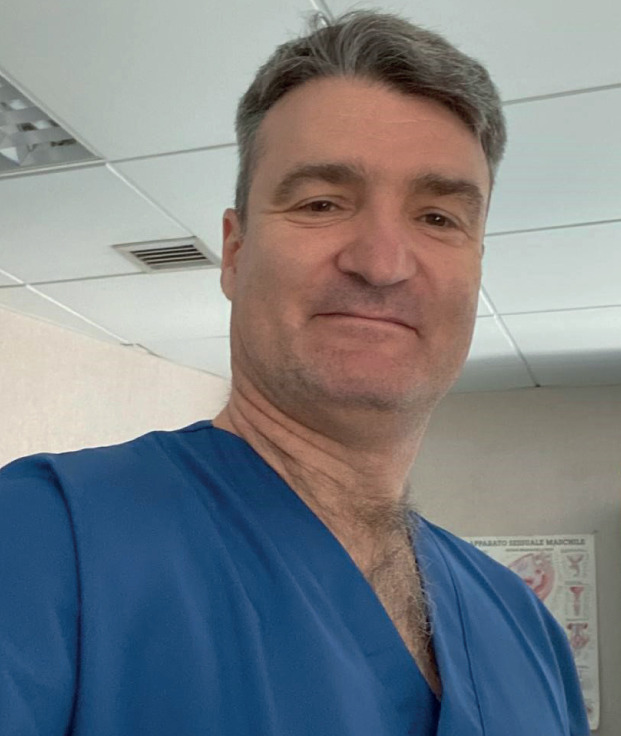


**Daniele Castellani**


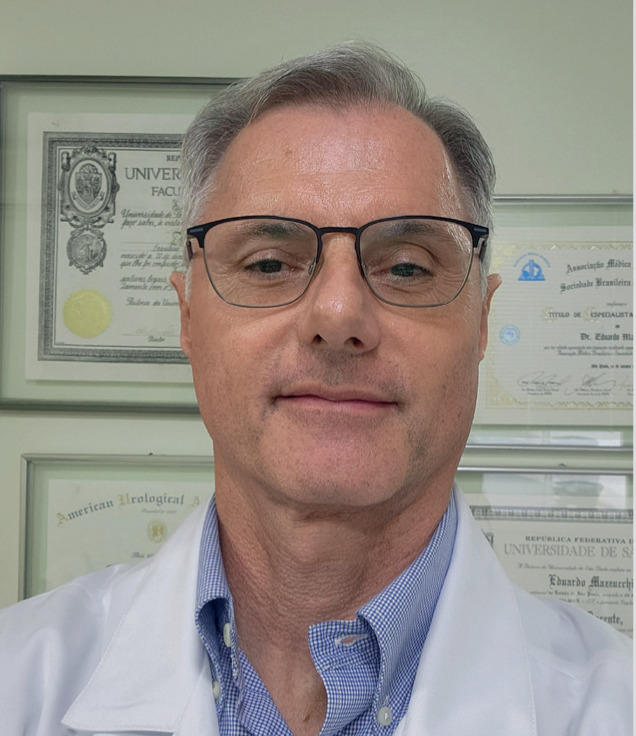


**Eduardo Mazzucchi**


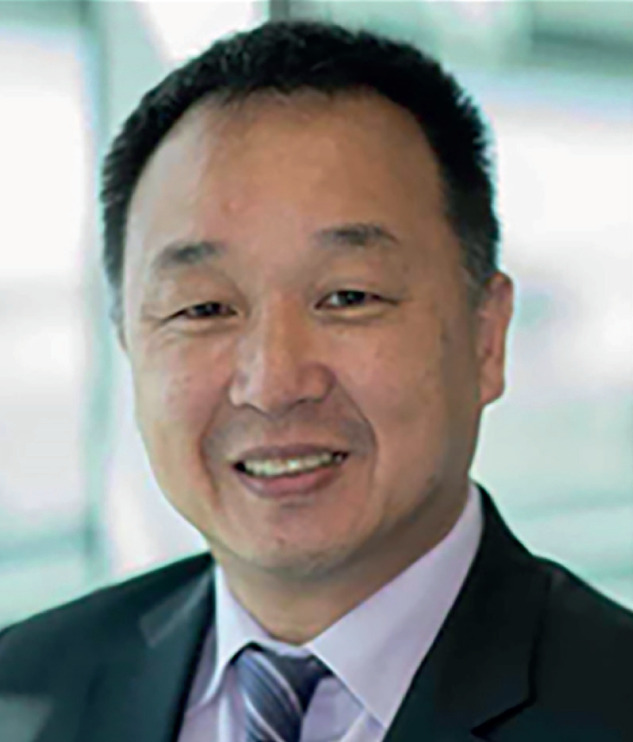


**Henry H. Woo**


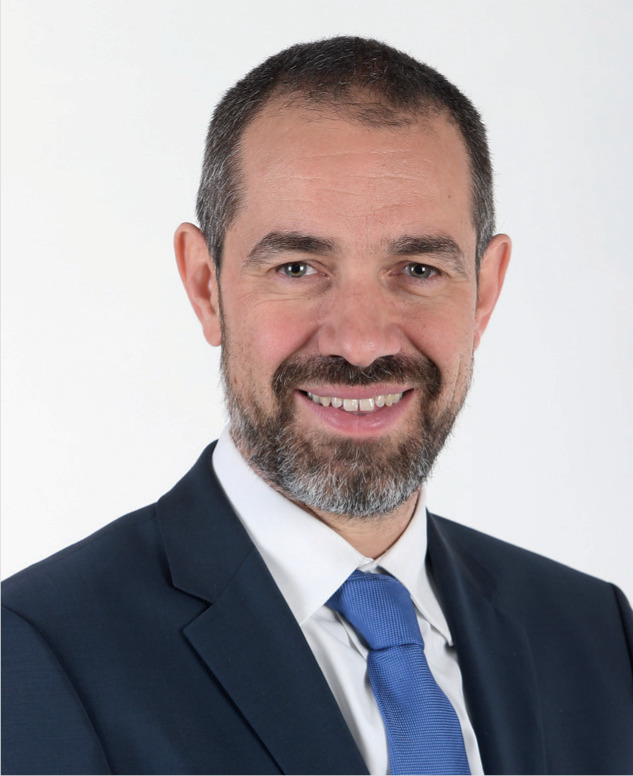


**Evangelos Liatsikos**


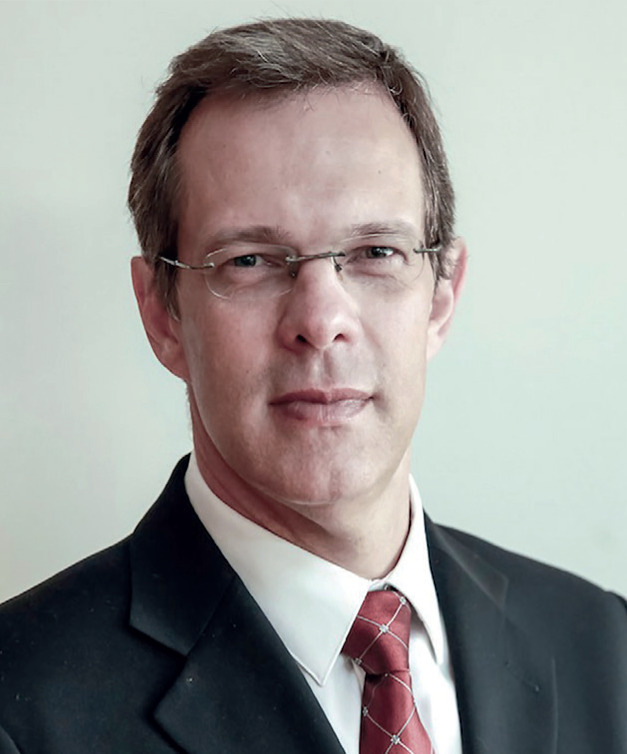


**José C. Truzzi**


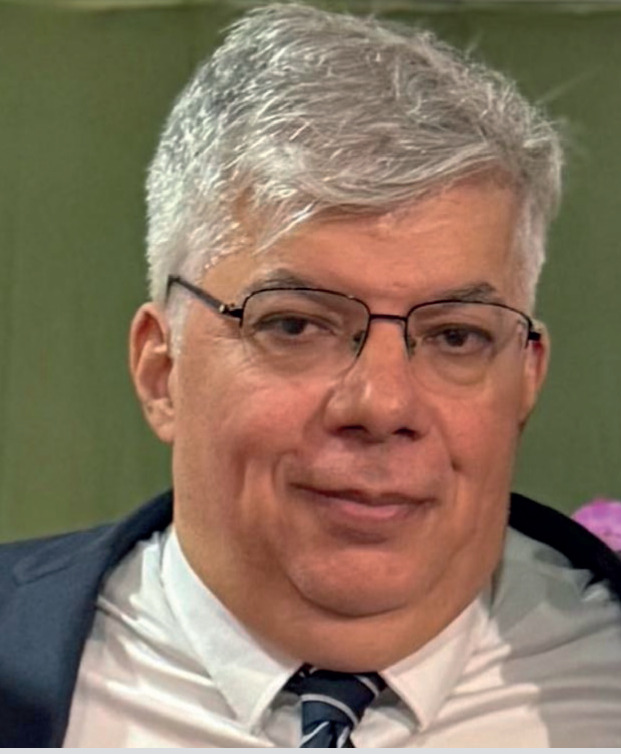


**José Bessa**


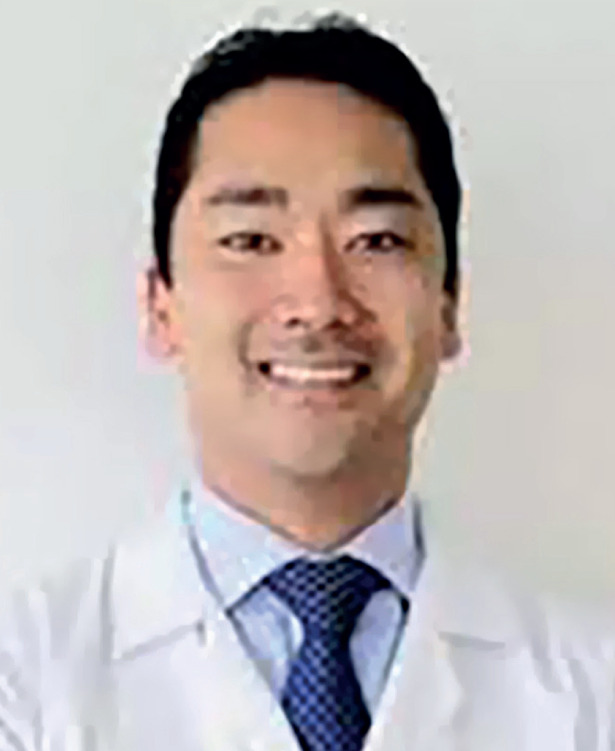


**Ricardo Miyaoka**


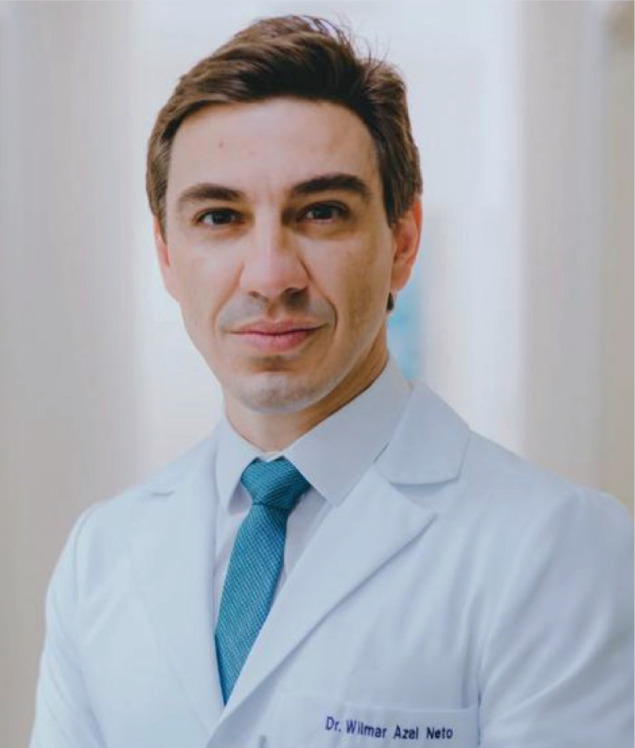


**Wilmar A. Neto**
